# Early developmental milestones in patients with idiopathic clubfoot treated by Ponseti method

**DOI:** 10.3389/fped.2022.869401

**Published:** 2022-08-24

**Authors:** Vito Pavone, Marco Sapienza, Andrea Vescio, Alessia Caldaci, Kathryn Louise McCracken, Federico Canavese, Gianluca Testa

**Affiliations:** ^1^Department of General Surgery and Medical Surgical Specialties, Section of Orthopaedics and Traumatology, P.O. “Policlinico Gaspare Rodolico”, University of Catania, Catania, Italy; ^2^Department of Pediatric Orthopedic Surgery, Jeanne de Flandre Hospital, Lille University Centre, Lille, France

**Keywords:** developmental milestones, motor milestones, linguistic milestones, idiopathic clubfoot, Ponseti

## Abstract

**Background:**

Idiopathic clubfoot, also referred to as congenital talipes equinovarus (CTEV), is one of the most common lower limb deformities observed in newborns, leading to significant functional impairment if untreated. The aims of this study were to (1) assess the developmental milestones in patients with CTEV treated by the Ponseti technique, and to compare them to the unaffected controls; (2) evaluate the possible correlation between developmental milestones, severity of the deformity, and number of casts.

**Materials and methods:**

Seventy-nine subjects were divided into two groups, CTEV group (43 patients; 72 feet) and control group (36 patients). Age, sex, affected side, attainment of babbles (BAL), independent gait (IG), and combined word (CW) were recorded for all patients. In patients with CTEV, Pirani Score (PRS), number of casts (NC), and clinical outcome were collected according to the Clubfoot Assessment Protocol (CAP), The American Orthopedic Foot and Ankle Score (AOFAS), and Foot and Ankle Disability Index (FADI).

**Results:**

IG was achieved later later than the unaffected controls by 12/43 patients (27.9%) with CTEV and 3/36 patients in the control group (8.3%) (*p* = 0.04) and in a mean time of 16.8 ± 3.5 months and 13.2 ± 2.7 months, respectively (*p* = 0.001). In the CTEV group the mean value of CAP was 98.6 ± 4.7, of AOFAS of 98.4 ± 4.4 and of FADI equal to 99.9 ± 0.44. There were no statistically significant differences for BAL and CW; and no correlation with PRS, NC, or clinical score were identified.

**Conclusion:**

CTEV patients managed by the Ponseti technique achieve independent gait later than the unaffected controls, although they do so within the age limit of developmental. On the other hand, the Ponseti treatment has no impact on attainment of language development.

## Introduction

Idiopathic clubfoot, also referred to as congenital talipes equinovarus (CTEV), is one of the most common lower limb deformities observed in newborns, leading to significant functional impairment if untreated (1, 2). Untreated or neglected CTEV may cause substantial disability due to foot deformity, pain, stiffness, and subsequent gait disturbance (1, 3, 4). Although multiple treatment options are available (5, 6), the Ponseti technique has become the most widely accepted protocol to treat CTEV during infancy. The technique is based on the application of an above-the-knee cast which corrects the foot gently and gradually; the cast is renewed every 5–7 days until the foot reaches between 40° and 50° of abduction in the transverse plane. At the end of cast treatment, up to 95% of patients require a percutaneous Achilles’ tenotomy in order to correct the residual equinus. This can be done either under general or local anesthesia. Following Achille’s tenotomy the foot is casted for an additional 2–3 weeks. Subsequently, the patients wear a foot abduction orthosis (FAO) to hold the foot in external rotation and dorsiflexion for 23 hours/day during the first 3–4 months, and then at night and during nap time until age 5 years (7–10). Several studies have reported patients with CTEV treated by the Ponseti technique with long-term outcomes (7, 10). In addition, more recent research studies have shown that patients treated by the Ponseti technique can achieve superior sports performance compared to patients managed by other techniques (8, 11). Moreover, in the comparison between male and female patients with uni- or bilateral CTEV involvement, functional results and similar sports performance were reported (11).

Overall, the Ponseti technique aims to achieve a pain-free supple and plantigrade foot with minimal surgery. However, there is a paucity of research investigating the gross motor skills (GMS) in children with CTEV managed by Ponseti technique (12, 13).

The aims of this study were to (1) assess the developmental milestones in patients with CTEV treated by the Ponseti technique, and to compare them to unaffected control; (2) evaluate the possible correlation between developmental milestones, severity of the deformity, and number of casts. The study hypotheses were the following: (a) the Ponseti treatment can delay early GMS but not linguistic skills compared to the normal population; (b) severity of the deformity and number of casts are not correlated to developmental milestones.

## Materials and methods

### Study design

The trial was conducted according to the Strengthening the Reporting of Observational Studies in Epidemiology (STROBE) Statement: guidelines for reporting observational studies (14). This prospective cross-sectional study was conducted according to the guidelines of the Declaration of Helsinki and approved by the Institutional Review Board A.O.U. Policlinico “G. Rodolico-San Marco” of Catania (protocol code 117/2020/PO, October 14, 2020).

### Sample eligibility criteria

Between January 2010 and June 2021, 104 patients were managed according to the Ponseti protocol at our institution.

The inclusion criteria were as follows: (1) a diagnosis of idiopathic CTEV at birth managed by Ponseti technique; (2) complete clinical data; (3) follow-up data spanning more than 2 years.

**FIGURE 1:**
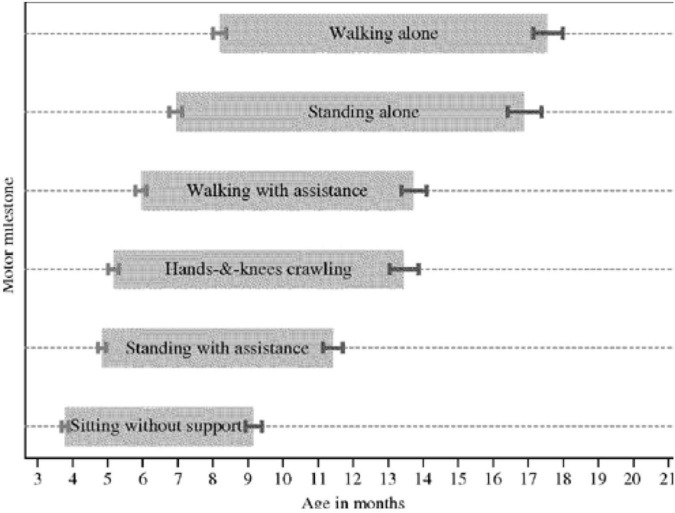
Study flow-chart.

Exclusion criteria were positional clubfoot, non-idiopathic CTEV, prior failed treatment, initial treatment at other Institutions, and clinical follow up of less than 2 years. A total of 43 of 104 patients (41.3%; 77 girls and 100 boys) met the inclusion criteria; the remaining 61 patients (59.7%) were excluded (Figure 1).

Additional information such as severity of the deformity according to the Pirani classification system (PRS), number of casts (NC), and the need of Achille’s tenotomy were retrieved from the charts (Table 1).

**TABLE 1:** Mean PRS and NC.

Group	PRS	Number of casts
CTEV	5.2 ± 0.88	7.1 ± 1.8
*PCC*	−0.1	−0.1
	0.46	0.51

Thirty-six unaffected children of similar age without CTEV or any other musculoskeletal disorder were included in the study as control group.

All guardians provided an informed consent to have their child participate in the present investigation.

### Treatment protocol and follow-up

All patients were treated according to the Ponseti technique and its principles (15). Long-leg cast was changed every 5–7 days, and 7 casts/patient were applied on average (range, 3–11); 43 out of 43 patients (100%) underwent percutaneous Achilles tenotomy under general anesthesia. FAO was prescribed until 5 years of age (15). Clinical follow up was performed every 6 months until 6 years of age and one a year thereafter.

### Primary outcome: Developmental milestones

#### Gross motion milestone assessment

Gross motor skills were investigated at each follow-up, since the patient was 4–5 months of age. Illustrations and demonstrations were provided to clarify the GMS when needed. The age of milestone achievement to the nearest month and the number of interviews per parent were recorded. In particular, as soon as the patient was able to do 10 consecutive steps without support, they were considered to have acquired IG.

**FIGURE 2:**
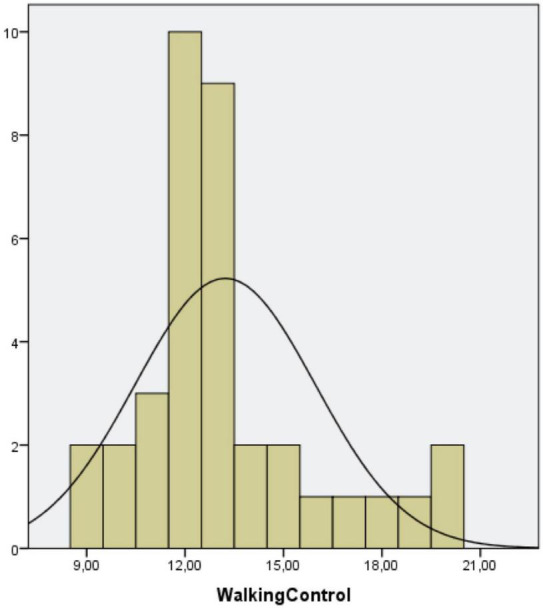
WHO motor development study: windows of achievement for six gross motor development milestones.

The windows of achievement for six gross motor development milestones (Figure 2) described by the World Health Organization (WHO) Multicenter Growth Reference Study Group were chosen to identify the proper cut-off limits (16).

#### Speech milestone assessment

Speech and language developmental milestones are noted in Table 2 and were investigated at each follow-up visit, since the patient was 4–5 months of age (Table 2). In particular, guardians were asked when their child achieved babbles (says “mama” or “papa”) and started to combine words.

**TABLE 2:** Developmental milestones for speech and language in children.

Age	Receptive	Expressive
6 months	Turns to rattling sound Turns to voice	Laugh* Vocalizes (cooing)*
9 months	-	Babbles, singles syllables* Says “mama” or “papa” non-specificallyΔ Waves “ciao.”Δ
12 months	Follows one-step command	Babbles* Imitates vocalizations and sounds* Says one wordΔ Waves “ciao”Δ
15 months	-	Says one word Says three wordsΔ Waves “ciao”*
18 months	Point to at least one body part	Says three words* Says six wordsΔ
2 years	Points to two pictures Follow two step commands	Combine wordsΔ Names one picturesΔ
2.5 years	Point to six body parts	Knows two actions.Δ Names one pictures* Speech half understandableΔ
3 years	-	Knows two adjectivesΔ Names four pictures* Names one colorΔ Speech all understandableΔ
4 years	-	Define wordsΔ Names four colorΔ Speech all understandable*

*More the 90% of children pass this item.

Δ50–90% of children pass this item.

### Secondary outcome: Congenital talipes equinovarus severity, number of casts, and clinical assessments

The severity of the clubfoot was assessed according to PRS by a senior pediatric orthopedic surgeon at the first clinical examination. The number of casts was retrieved from medical charts. Clinical and functional outcomes of all patients were evaluated in patients with at least 2 years of follow-up, using the Clubfoot Assessment Protocol (CAP) (17), the American Orthopedic Foot and Ankle Society (AOFAS) Ankle–Hindfoot score (18), the Foot and Ankle Disability Index (FADI) (19). The PRS, CAP, AOFAS, FADI scores in patients with bilateral involvement are the mean between the CTEV.

### Pirani score

The PRS includes the presence and the severity of six items: medial crease (MC-PRS), lateral part of the head of talus, curvature of the lateral border, posterior crease, empty heel, and rigid equinus. Each of the six items is scored on a three-point scale (0 = none, 0.5 = moderate, 1 = severe abnormality). The total score ranges from 0 to 6 based on the severity of the deformity (20).

### Clubfoot assessment protocol

The CAP contains 22 items in four sub-groups: mobility (8 items), muscle function (3 items), morphology (4 items), and motion quality I and II (7 items). The first three sub-groups relate to body function/structures and the last relates to activity. These groups include standardized questions about pain, stiffness, and daily activity/sport participation. The scoring is divided systematically and proportionally with respect to normal variation and its supposed impact on perceived physical function ranging from 0 (severe reduction/no capacity) to 4 (normal). Score grading varies from 3 to 5 levels. For each subgroup, the sum of the item scores are calculated and visualized as profiles (converted to a 0–100 scale score, with 0 = extremely deviant and 100 within normal variance; sub-group transformation score = actual score/maximal possible score × 100) (17).

### American orthopedic foot and ankle society Ankle–Hindfoot

The AOFAS Ankle-Hindfoot score consists of nine items under three different categories, i.e., pain (40 points), functional aspect (50 points), and alignment (10 points), totaling 100 points. Items on pain and functional limitation are answered by the patient, while the alignment items are answered by an examiner (18).

### The foot and ankle disability index

Foot and ankle disability index is a region-specific self-reporting scale of function that includes 34 items divided into two subscales, the first (FADI) consisting of 26 items concerning activities of daily living (ADL) and pain, and the second (FADI Sport) consisting of eight items concerning sports activities. Each item is scored on a five-point Likert scale (0–4) and, thereafter, the results are converted into percentages. Higher scores represent higher levels of function for each subscale. The ADL and Sport subscales are scored separately (21).

### Statistical analysis

Continuous data are presented as means and standard deviations, as appropriate. The student’s *t*-test was used to evaluate the mean and standard deviation between groups. The Chi-square test was used to verify the homogeneity of the group and to compare number of patients who achieved the milestone before and after the upper limit milestones. The selected threshold for statistical significance was *p* < 0.05. The Pearson Correlation Coefficient (PCC) was utilized to assess the correlation between the walking alone milestone and PirS or number of casts. The PCC is a value between −1 and 1, where values close to −1 indicate high negative correlation, values close to 1 indicate high positive correlation, and values close to 0 indicate no or very week correlation. The correlation coefficient is rated according to Colton (22):

• 0 to 0.25 (0 to −0.25) little or no relationship;
• 0.25 to 0.50 (−0.25 to −0.50) fair degree of relationship;
• 0.50 to 0.75 (−0.50 to −0.75) moderate to good relationship;
• 0.75 to 1.00 (−0.75 to −1.00) very good to excellent relationship.

All statistical analyses were performed using IBM SPSS, Version 24.0 (IBM Corp., Armonk, NY, United States).

## Results

### Sample

The overall cohort of patients consisted of 42 males (53.2%) and 37 females (46.8%) with a mean age of 8.4 ± 3.9 years (range, 2–17).

Forty-three children (74 feet) were treated with the Ponseti technique and composed the CTEV group [27 males and 16 females; mean age 7.8 ± 3.6 years (2–14); mean follow-up 9.3 ± 5.7 years (0–14)], while 36 unaffected children composed the control group [15 males and 21 females; mean age 9.1 ± 4.7 (2–17); mean follow-up 6.8 ± 8.7 (0–16)] (Table 3). The mean age of the two groups were comparable (*p* > 0.05).

**TABLE 3:** Demographics.

Group	Patients	Gender		Mean age (Years)	Mean follow-up (Years)
		Male	Female		
CTEV	43	27	16	7.8 ± 3.6 (2–14)	9.3 ± 5.7 (0–14)
Control	36	15	21	9.1 ± 4.7 (2–17)	6.8 ± 8.7 (0–16)
*p*		0.06		0.17	0.13

### Developmental milestones

**FIGURE 3:**
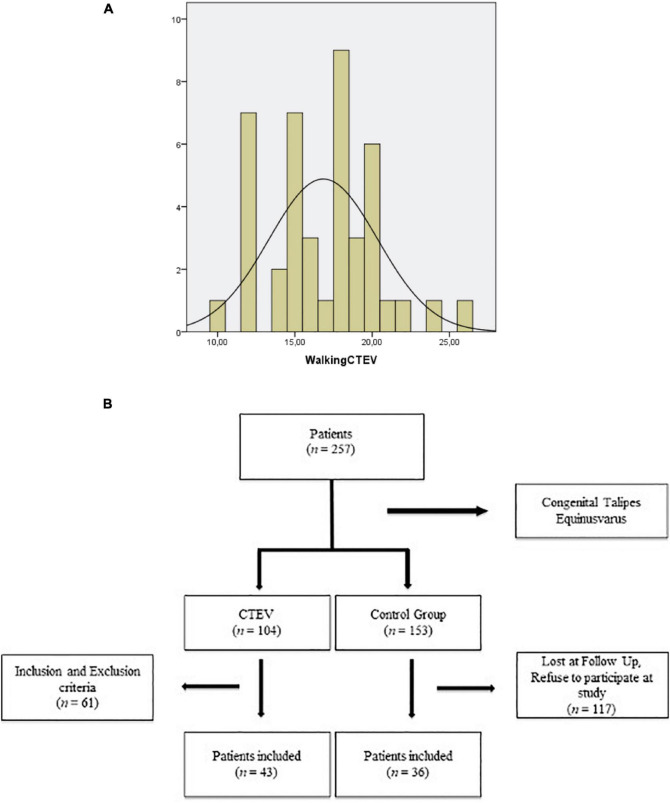
**(A)** Distribution of patients with CTEV achieving IG. **(B)** Distribution of unaffected patients achieving IG.

**FIGURE 4:**
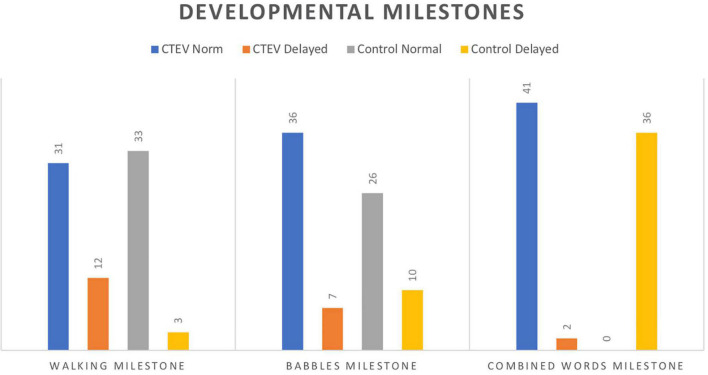
Developmental milestones.

Statistically significant differences were found regarding the number of patients achieving independent gait after the age of 18 months according to the WHO Multicenter Growth Reference Study Group Cut-off: CTEV Group (12/43 patients; 27.9%) vs. control group (3/36 patients; 8.3%) (*p* = 0.04) (Figures 3A,B) as well as in the average number of months required to achieve independent gait: CTEV Group: 16.8 ± 3.5 months vs. control Group: 13.2 ± 2.7 months (*p* = 0.0001) (Table 4 and Figure 4).

**TABLE 4:** Developmental milestones.

Group	Independent gait (patients)	Independent gait acquisition (mean; months)	Babbles (patients)	Babbles (mean; months)	Combined words (patients)	Combined words (mean; months)
CTEV	12/43	16.8 ± 3.5	7/43	7.7 ± 2.0	2/43	18.9 ± 4.5
Control	3/36	13.2 ± 2.7	10/36	8.3 ± 2.1	0/36	18.5 ± 5.2
*p*	0.04	0.0001	0.3	0.20	0.5	0.71

Speech and language developmental milestones (babbles) were achieved by 7/43 patients of CTEV group (16.3%) and by 10/36 patients in control group (27.8%) (*p* = 0.3), after the age of 10 months, with no statistical differences for the mean time needed to achieve the milestone: CTEV Group: 7.7 ± 2.0 months vs. control Group: 8.3 ± 2.1 months (*p* = 0.2).

Comparable number of patients did not achieve the “combine words” stage by the age of 24 months. In particular, 2/43 (4.7%) patients in CTEV group vs. 0/36 patients in control group (*p* = 0.5). Similarly, the average number of months needed to reach the “combined words” stage [CTEV Group (18.9 ± 4.5 months) vs. control group (18.5 ± 5.2 months); *p* = 0.71] was comparable (Table 4).

### Secondary outcome

#### Clubfoot severity according to Pirani Score

**FIGURE 5:**
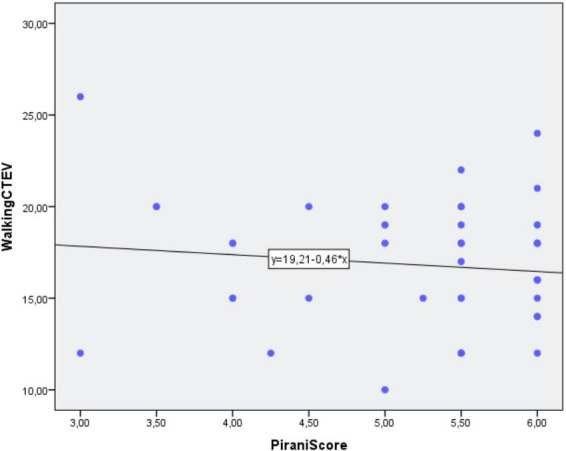
Correlation between independent gait and Pirani score.

The mean PRS of affected children was 5.2 ± 0.88. The PCC between the two groups for the number of months required to reach independent walking and PRS was not statistically significant (PCC = –0.1; *p* = 0.46) (Figure 5 and Table 5).

**TABLE 5:** Correlation between CTEV severity, number of casts, and outcome.

Group	PRS	Number of casts	CAP	AOFAS	FADI
CTEV	5.2 ± 0.88	7.1 ± 1.8	98.6 ± 4.7	98.4 ± 4.4	99.9 ± 0.44
*PCC*	−0.1	−0.1	0.2	0.3	0.2
	0.46	0.51	0.12	0.58	0.17

PCC, Pearson’s correlation coefficient; PRS, Pirani Score; CAP, Clubfoot Assessment Protocol; AOFAS, American Orthopedic Foot and Ankle Society Ankle-Hind foot Score; FADI, Foot and Ankle Disability Index.

#### Number of casts

**FIGURE 6:**
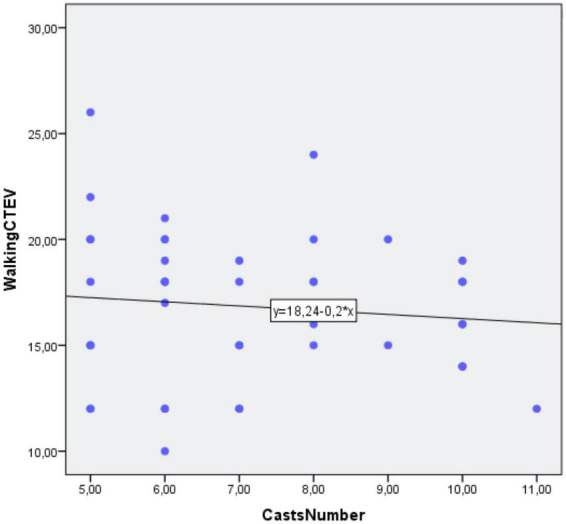
Correlation between independent gait and Number of Casts.

The mean number of casts was 7.1 ± 1.8 (range, 3–11). The PCC between the two groups for the number of months needed to reach the independent gait stage and number of casts was not statistically significant (PCC = −0.1; *p* = 0.51) (Figure 6 and Table 5).

#### Clinical assessments: Clubfoot assessment protocol, the American orthopedic foot and ankle score, and foot and ankle disability index score

**FIGURE 7:**
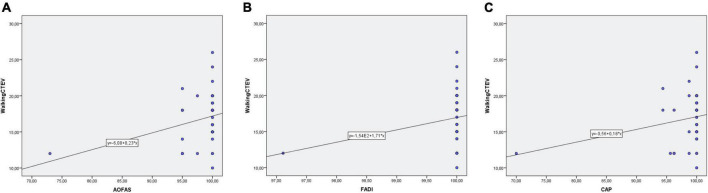
**(A)** Correlation between independent gait and CAP Score. **(B)** Correlation between independent gait and AOFAS. **(C)** Correlation between independent gait and FADI Score.

The mean CAP was 98.6 ± 4.7. The PCC between the two groups for the number of months needed to reach independent gait and CAP was not statistically significant (PCC = 0.2; *p* = 0.12) (Figure 7A).

Mean AOFAS recorded was 98.6 ± 4.7. The PCC between the two groups for the number of months needed to reach independent gait and CAP was not statistically significant (PCC = 0.3; *p* = 0.58) (Figure 7B).

The mean FADI recorded was 98.6 ± 4.7. The PCC between the two groups for the number of months needed to reach independent gait and CAP was not statistically significant (PCC = 0.2; *p* = 0.17) (Figure 7C).

## Discussion

This study found that patients with CTEV treated according to the Ponseti technique, tend to acquire independent gait later (3 months) than unaffected children (Figures 3A,B and Table 4).

Sala et al. (23) found the delay for independent gait averaged about 2 months with half of the children walking at 13.8 months and nearly all at 17.7 months of age (23).

Karol et al. (12) reported the majority of children with CTEV evaluated with the Peabody Developmental Gross Motor Scale at age 5 years had average gross motor function. The Peabody Developmental Gross Motor Scale assesses whole body development and motor ability, but does not specifically evaluate the quality of lower limb movements. For example, it merges the ability to stand on one foot and to perform sit-ups in the same domain (12).

Andriesse et al. (24) reported a prevalence of 35% of motor impairments in the children aged 7 years with CTEV using the Movement Assessment Battery for Children (24). Alternatively, Lööf al. (13) reported most children with CTEV had difficulties with toe and heel walking and standing and hopping on one foot when patients were evaluated at age 5 years.

However, such delays in developmental milestones are not expected to have a lasting effect on the child’s development. In fact, most children with CTEV have comparable motor skills and can participate in sports without any problems by 9 years of age, as reported by Hughes et al. (25). Therefore, it is more appropriate to look at the age limit of development, which denotes the age by which all children should have achieved a commonly accepted milestone. For walking, the age limit of development is 18 months (26). In the study by Hughes et al., 93% of children with CTEV achieved ambulation by 18 months (25). Therefore, although affected children may be slightly delayed compared to unaffected individuals (17.7 months vs. 13.8 months in our cohort), they will still achieve motor development milestones well within the age limit of the healthy population (18 months).

The Ponseti Technique implies the use of foot and ankle orthosis (FAO) up to the age of 5 years. Therefore, it is possible that the use of FAO may have a clinical impact on the acquisition of independent gait through persistent immobility. The mild delay observed in children with CTEV is likely to be multifactorial, due to a combination of primary pathological factors, secondary treatment factors, and cultural and behavioral factors. FAO compliance was monitored carefully as patients were always assessed by the same surgeon. It is also important to recognize that compliance with FAO is an important predictor of later recurrence (7). Compliance with FAO is unlikely to have had a clinical impact on other developmental milestones (25). According to our results, the two groups of patients had similar speech and language developmental milestones (babbles and “combined words”).

There were some limitations in the analysis of our results. Parental recall of developmental milestones data may be subject to inaccuracy. However, Majnemer et al. found that at age three there was a greater than 70% correlation between parental recall and walking age (27). Secondly, the number of patients enrolled is relatively low. However, all patients were treated and followed up by the same surgeon according to the same protocol. In addition, the demographics of the two groups of patients were comparable, as well as the length of follow-up (Table 3). Lastly, 86.2% of children included in the study had bilateral clubfoot; this should be considered as an additional statistical bias.

## Conclusion

Children with CTEV treated by the Ponseti technique show slight developmental delays in reaching independent gait compared to unaffected children. Despite this, they still achieve the milestone within normal time limits and functional outcome is generally good. On the other hand, Ponseti Treatment has no impact on the attainment of language development. Future prospective, randomized trials with a larger sample size of CTEV patients are now needed to confirm the findings of our study.

## Data availability statement

The raw data supporting the conclusions of this article will be made available by the authors, without undue reservation.

## Ethics statement

Ethical review and approval was not required for the study on human participants in accordance with the local legislation and institutional requirements. Written informed consent to participate in this study was provided by the participants’ legal guardian/next of kin.

## Author contributions

VP, GT, AV, and MS treated the orthopedic aspect. MS and KM contributed to early developmental milestones topic. FC revised the manuscript. All authors listed have made a substantial, direct, and intellectual contribution to the work, and approved it for publication.
